# 1103. Case Study. *Lichtheimia Ramosa*: Emerging Virulent Fungal Infection in Burn Wounds and Effective Treatment Case Study

**DOI:** 10.1093/jbcr/irag033.207

**Published:** 2026-04-06

**Authors:** Chloe Jensen, Alyssa D Brown

**Affiliations:** Texas Tech University Health Sciences Center, Lubbock, TX; Texas Tech University Health Sciences Center, Lubbock, TX

## Abstract

**Patient Presentation (age range, injury details, relevant history):**

36 year old female 60% TBSA burn was admitted to our service. Initial treatment was debridement, biodegradable temporizing matrix, and autografting. This resulted in failed grafts due to infection. Signs of wound infection were present and lab tests confirmed the presence of Lichtheimia on skin biopsy.

**Clinical Challenges:**

Mucor species are becoming more frequently encountered in burns secondary to their ubiquitous nature in the environment. While cutaneous mucor is more likely to occur in immunocompromised patients overall, trauma and large burns can also be infected by Mucor species. Lichtheimia is emerging as an increasingly common cause of cutaneous fungemia. Lichtheimia species of Mucor are more likely to transform into disseminated infections, likely due to their pro-angiogenic properties. We present a case study of a patient with persistent cutaneous Lichtheimia resulting in multiple graft failures and successful treatment with a combination of longer duration of topical amphotericin B and aggressive debridement.

**Management Approach:**

To treat the infection, we applied laparotomy pads soaked in amphotericin b at twelve hour intervals over the burn sites for a duration of 7 days. The patient had no conversion to systemic fungemia following extended treatment with amphotericin b and did not require systemic antifungal therapy.

**Outcomes:**

After clearance of the cutaneous lichtheimia, the patient had successful grafting with acellular fish xenograft followed by autograft without any further positive cultures for lichtheimia. The patient has been successfully discharged to inpatient rehabilitation.

**Lessons Learned:**

Mucor is frequently found in soil and organic plant debris. Mucor infections can quickly spread from cutaneous manifestations to disseminated fungemia. Lichtheimia species of mucor is more virulent than other mucor species leading to a higher rate of conversion to disseminated fungemia. Major burns are especially sensitive to these fungal infections leading to increased morbidity and mortality. Aggressive treatment with topical amphotericin b can decrease the conversion of these cutaneous lichtheimia infections into disseminated fungemia. This method avoids systemic antifungals, which have their own morbidities. Burns could be successfully re-grafted following successful clearance of cutaneous Lichtheimia infection.

**Applicability to Practice:**

Speciation of Mucor species can identify more pathogenic strains, like lichtheimia, and may require more aggressive treatment achieved through a combination of longer treatment with topical amphotericin b, avoiding systemic antifungal treatment.

